# Spatiotemporal patterns of antimicrobial resistance in shared human-animal waterpoints in the Maasai Mara Ecosystem, Kenya

**DOI:** 10.1371/journal.pgph.0007393

**Published:** 2026-10-07

**Authors:** Brian Marvis Waswala-Olewe, George Paul Omondi, Monica Adhiambo Olewe, Brian Njuguna, Yvonne Nyararai, Nyamai Mutono, Mwangi Thumbi, Paul W. Webala, Romulus Abila

**Affiliations:** 1 Department of Environmental Studies, Geography and Planning, Maasai Mara University, Narok, Kenya; 2 Kenya National Commission for UNESCO, Nairobi, Kenya; 3 Baruk Yadiym Ecosphere, Nairobi, Kenya; 4 Centre for Epidemiological Modelling and Analysis, University of Nairobi, Nairobi, Kenya; 5 World Health Organization, Kampala, Uganda; 6 Department of Animal and Wildlife Sciences, Midlands State University, Gweru, Zimbabwe; 7 Paul G. Allen School for Global Health, Washington State University, Washington, United States of America; 8 Institute of Immunology and Infection Research, University of Edinburgh, Edinburgh, United Kingdom; 9 Department of Forestry and Wildlife Management, Maasai Mara University, Narok, Kenya; University of Oslo Faculty of Medicine: Universitetet i Oslo Det medisinske fakultet, NORWAY

## Abstract

Antimicrobial resistance (AMR) presents an existential One Health threat. The spread of AMR in arid and semi-arid landscapes is modulated by ecological interactions and exacerbated by endemicity of livestock diseases and poor sanitation in shared human-animal systems leading to misuse of antibiotics. This study characterized the prevalence, resistance patterns, and spatiotemporal variations of AMR in surface water sources in the Maasai Mara Ecosystem (MME), a rapidly changing water-stressed socio-ecological landscape. Employing cross-sectional study design, water samples were collected from 28 water points between April 2024 and May 2025. Cultured *Escherichia coli* isolates were evaluated for resistance to eight antibiotics, following CLSI susceptibility testing protocols. AMR prevalence was calculated as proportions, with associations between sampling sites and seasons assessed using chi-square. Local clustering of resistant isolates was evaluated using spatial multinomial modelling, while cost-distance analysis against water points identified the proportion of the MME population with limited access to potable water. *E. coli* was detected in 92.9% water sources, with all isolates resistant to at least one antibiotic. High resistance rates were observed for Penicillin (100%), Amoxicillin (92.6%), Nitrofurantoin (78.6%), and Ceftazidime (52.9%). River samples exhibited higher resistance (51.4%) than open water pans (40.4%). AMR peaked during short-wet and short-dry seasons (51.3%; 51.5% respectively), with seasonal variations being significant (*p = 0.04*). Multi-drug-resistant isolates (MDR: resistant to ≥3 antibiotic classes) were detected across all seasons, peaking in the short-wet season. MDR relationship between water sources was insignificant across all sampling sites and seasons (*p > 0.05*). Spatial analysis (*p > 0.05*) identified two exploratory clusters: Cluster 1 near dense human areas (RR = 0.78) while Cluster 2 in human-livestock-wildlife zones (RR = 1.26). Cost-distance analysis (*p < 0.05*) revealed one-third of the MME population is at risk of AMR due to limited access to potable water. Our findings reveal widespread distribution of AMR isolates in shared waterpoints, necessitating targeted One Health Quadripartite interventions.

## Introduction

Antibiotics play a crucial role in preventing and treating infections, thereby improving health outcomes for both humans and animals, as well as contributing to food production [1,2]. However, antimicrobial resistance (AMR) is becoming a significant global public health challenge [3–6], with an estimated five million human fatalities attributed to AMR in 2019. It is projected that ten million deaths will result from AMR by 2050 [7,8], particularly in Africa and South Asia, where inadequate water, sanitation and health infrastructure, alongside multi-use human-animal landscapes are common [9–11].

Kenya is a hotspot for antimicrobial resistance emergence due to the poor stewardship and use of antimicrobials in both humans and animals [12–16]. Shared human-animal environments often amplify these challenges, especially in rangeland ecosystems that host large numbers of livestock, wildlife, and humans, coupled with widespread antimicrobial use and environmental residues that enhance selection pressure [17,18]. The misuse of antimicrobials, particularly in pastoral environments, is driven by the high incidence of endemic livestock diseases owing to sympatry between livestock and wildlife, and limited access to veterinary care [16,19,20].

Surface water sources in these shared ecosystems frequently serve as sinks for waste from humans and animals, containing antibiotics, metabolites, and antibiotic-resistant bacteria, which have landscape-level health implications [18], perpetuating a transmission cycle for antibiotic resistant bacteria among humans, wildlife, livestock and the environment [21]**.** However, there is paucity of data on the occurrence and distribution of antimicrobial resistance in multi-use watering points, especially in arid and semi-arid lands (ASALS), alongside their associated risks hindering a cohesive “One Health Quadripartite” strategy to address the intersection of human, animal, and environmental health [22].

In Kenya, 60% of the population lacks access to potable water [23,24], and in Narok County, fewer than 10% have piped water [25,26]. Most residents rely on shared surface water sources susceptible to contamination by pathogens such as *Escherichia coli* (*E. coli*), *Shigella, Proteus, Salmonella*, *Enterobacter*, *Klebsiella*, and *Citrobacter* species, rendering these water sources unfit for consumption [27–29]. The World Health Organization (WHO) identifies *E. coli,* a gram-negative bacterium, as a key indicator of fecal contamination in water [30]. *E. coli* is a priority pathogen for antimicrobial resistance surveillance nationally and globally [31,32]. Rising resistance increases morbidity, mortality, lengthens hospitals stays, and treatment costs, livestock losses and productivity and food safety Poor sanitation and open defecation worsen contamination and drive proliferation of AMR in shared water systems [33–36]. Narok County faces growing climate variability leading to increasing water scarcity and dependence on surface water sources, which necessitates pathogen risk assessments [26,37–39]. The Maasai Mara Ecosystem (MME), part of the Mara–Serengeti landscape, is a major tourist destination [40] linked to potential spread of travel-related *E. coli* [41,42]. Additionally, the seasonal migration of livestock, humans, and wildlife, along with the close interactions between livestock and wildlife [43], may further amplify transmission of waterborne pathogens, including AMR isolates [19,44].

To manage the emerging AMR threat, there is a need to assess the occurrence and distribution of AMR isolates in these shared water systems as a precursor to developing targeted One Health interventions [45,46]. This study aimed to characterize AMR prevalence, resistance patterns, and seasonal variations of *E. coli* isolates from surface water sources and evaluate spatial distribution of resistance profiles in the MME. Specifically, we sought to: i) determine the prevalence of AMR *E. coli* isolates in surface waters while analyzing trends in antimicrobial resistance, including resistance patterns for individual antibiotics; ii) assess seasonal variations in AMR *E. coli* isolates across sampling periods and evaluate differences in antibiotic resistance by water source; and iii) identify the spatiotemporal distribution of antimicrobial resistance profiles and risks to populations.

## Materials and methods

### Ethical approval

This study was approved by Egerton University Institutional Scientific and Ethics Review Committee (EUISERC/APP/327/2024) and the National Commission for Science, Technology, and Innovation in Kenya (NACOSTI/P/24/35318). These isolates were purely from environmental sources. To access water sources, verbal consent was obtained from civic leaders, local administration, and private land tenure owners.

### Study area

This study was conducted in Narok West subcounty, Narok County (1.3605° S, 35.7407° E), within the Greater Mara-Serengeti Ecosystem (Fig 1). The landscape’s ambient temperature ranges from 12 °C to 28 °C, and the bi-modal annual rainfall varies between 500 mm and 1,800 mm, influenced by the Intertropical Convergence Zone (ITCZ) and the topographic relief from Lake Victoria [47]. The predominant Maasai community is largely rural, engaging in livestock/animal pastoralism, wildlife tourism/conservation, and crop farming [48–50]. Wildlife conservancies, adjacent to the Maasai Mara National Reserve, serve as wildlife dispersal areas [37,38], with the reserve driving human and animal movement across the ecosystem [43]. The community herein has been shown to experience a high incidence of livestock diseases, with accompanying misuse of antibiotics [16,51] and limited access to veterinary care and increased antimicrobial resistance in humans and animals [19,20,52].

**Fig 1:**
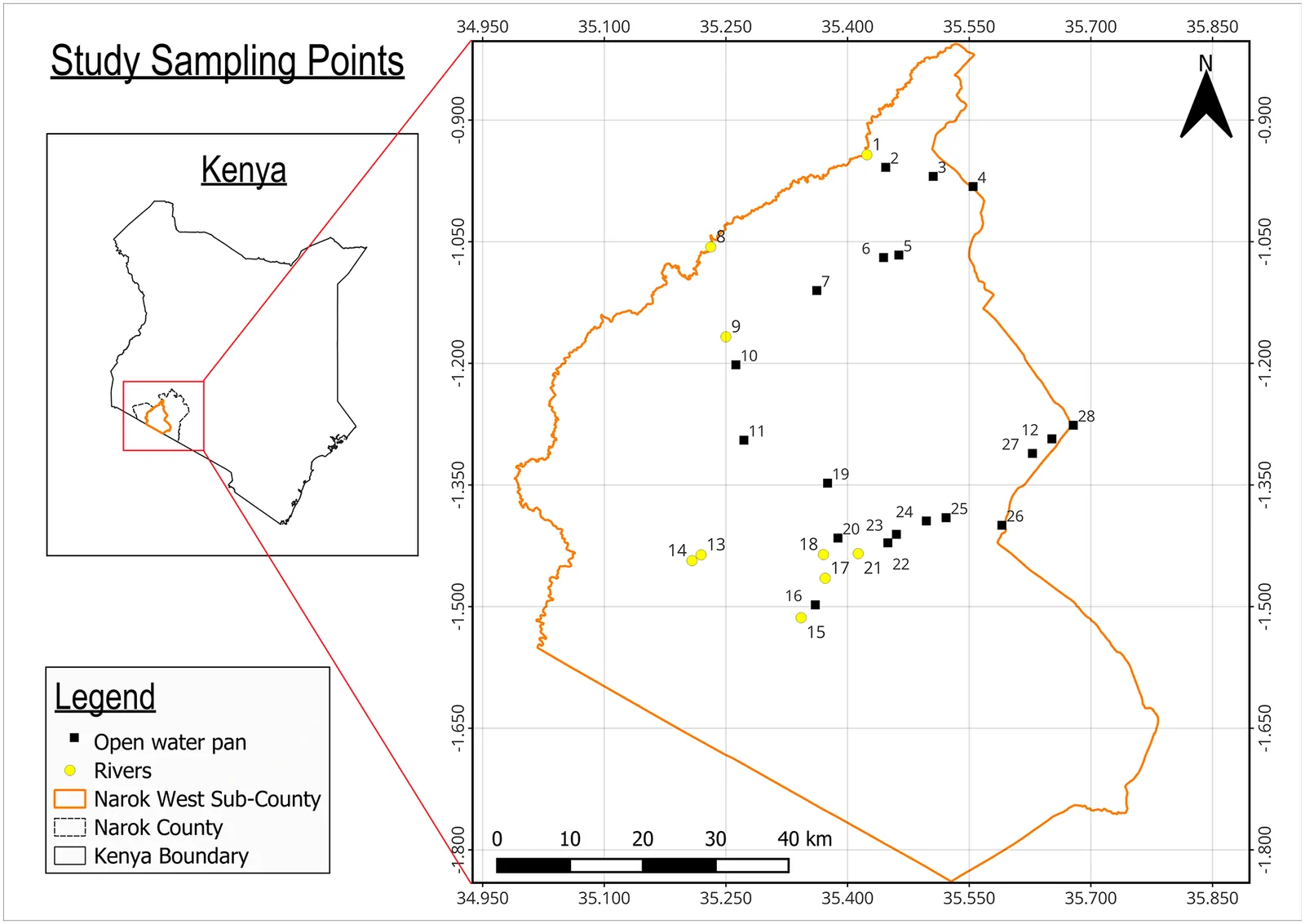
Map of Kenya showing location of Narok West subcounty, with sampled surface sources as points. Caption credit: Waswala-Olewe BM.

### Characteristics of sampling points and sample collection

The sampling sites consisted of open water pans and rivers. The primary water sources were open access and multi-use, accessible by both humans, livestock and wildlife, with notable presence of fecal matter on the banks (Fig 2; S1 Table). Open water pans (OWPs) were defined as natural or excavated depressions filled by water, commonly used by herders to water livestock, farmers for irrigation, water wildlife and other community members for domestic use. OWPs store surface runoff water and usually lose water through seepage and evaporation. Twenty-eight randomly selected waterpoints (eight river points and twenty open water pans) were sampled between May 2024 and April 2025. One water sample per site was collected seasonally (once every 3 months) over a period of 12 months spanning both dry and wet seasons. Metadata (geocoordinates and water feature—river vs. open water pan) were also recorded. Aseptic polyethylene bottles were used to collect surface waters at a depth of 10 – 15 cm below the water surface; and 20 – 30 centimeters from the open water pan shoreline / riverbank [53]. Caution was taken not to raise sediments when filling the bottles as sediments harbor settled microbes [54]. The samples were transported to Maasai Mara University Biology Laboratory in cooler boxes for analysis within 24 hours.

**Fig 2:**
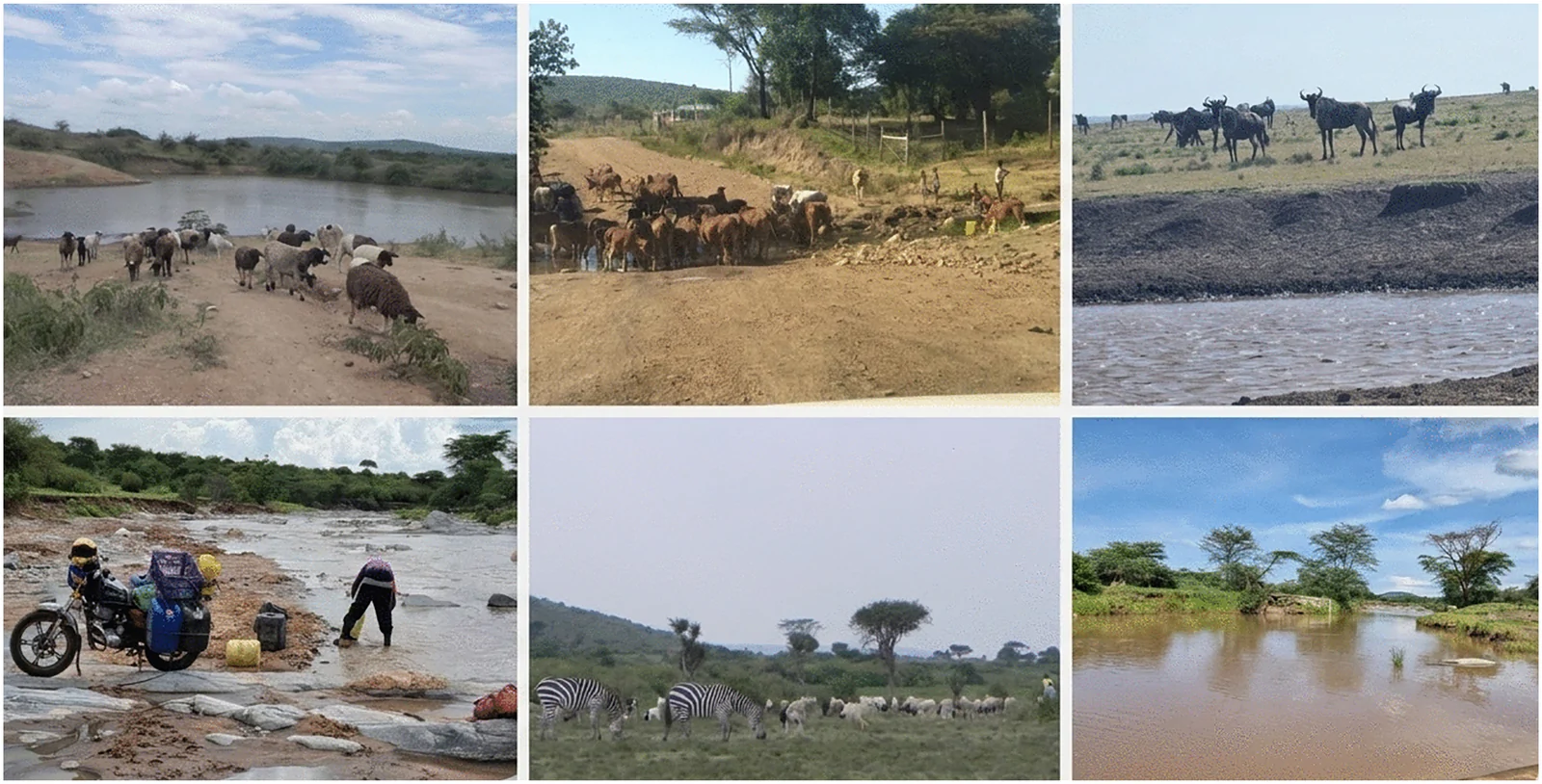
Multi-use surface water sources used by humans, livestock and wildlife in MME. 2A Open water pan used by livestock. 2B Sekenani River is used by humans and livestock. 2C Open water pan used by wildlife. 2D Ormirui River is used by humans, livestock and wildlife. 2E Shared human-livestock-wildlife landscape. 2F Talek River is used by humans, livestock and wildlife. Caption credit: Waswala-Olewe BM.

### Laboratory culture, and antimicrobial susceptibility testing

At the laboratory, using serial dilution, 50μl aliquots of water samples were filtered through sterile 0.45 μm cellulose nitrate membrane filters (Filtr8 Lab Vacuum Filtration Kit), then incubated on Chromogenic Coliform Agar (HiCrome™ CCA, Himedia, India) for 24 hours [46]. *E. coli* colonies were identified phenotypically [47–49], with one presumptive colony sub-cultured on MacConkey Agar (HiMedia M008S-500G, India) and incubated at 37°C for 24 hours. Following incubation, one confirmed *E. coli* isolate per site was evenly spread on Muller Hinton Agar (Himedia M173-500G, India) using sterile cotton swabs and allowed to stand for ten minutes. Eight antibiotic discs were tested: Ceftazidime 30 mcg (CAZ_30), Amoxycillin 30 mcg (AMX_30), Gentamicin 10 mcg (GEN_10), Levofloxacin 5 mcg (LVX_5), Nitrofurantoin 300 mcg (NIT_300), Tetracycline 30 mcg (TCY_30), Kanamycin 30 mcg (KAN_30), and Penicillin G 10/10 μg (PEN_10). Kenya’s Ministry of Health [15,55] and the World Health Organization (WHO) rank these antibiotics from important to highest priority and critically important [56], while the World Organization for Animal Health (WOAH) ranks them all as Veterinary Critically Important Antimicrobial Agents [57]. Plates were incubated at 37°C for 18 hours [58]. Antibiotic susceptibility was determined using the Kirby-Bauer disc diffusion method, following the Clinical & Laboratory Standards Institute [59] guidelines, and classified as susceptible, intermediate, or resistant.

### Data analysis

Data was entered into Microsoft Excel (Microsoft, Redmond, WA, USA) for analysis. Summary statistics were computed for sampling locations and seasonal trends, and chi-square tests were used to evaluate the associations between resistance profiles, seasons, and locations. Further, we evaluated multidrug resistance [60]. Multidrug resistance (MDR) was defined as resistance to three or more antibiotic categories.

#### Local cluster analysis.

Identification of clustering patterns was conducted by local cluster analysis. We hypothesized that the Maasai Mara National Reserve influences the spatial clustering of resistance profiles, modulating resource sharing among humans, livestock, and wildlife. A purely spatial multinomial model of the spatial scan statistics was performed using SaTScan v10.3.2 [60,61]. AMR isolates were geocoded based on their sampling site (unit of analysis), with three nominal attributes: category 1 (*Susceptible*), category 2 (*Intermediate*), and category 3 (*Resistant*). Using a likelihood ratio test, the purely spatial multinomial cluster analysis compares cases using a circular scanning window moving across the geocoded sampling sites’ space [62]. The windows are variable in size, and when a cluster is identified, a test statistic comparing the potential cluster with the remaining area is calculated based on a permutation approach, thus testing the hypothesis that similar antimicrobial profiles are more likely to occur within the clusters than expected by chance [61,62]. The windows that maximize the likelihood ratios, which compare the observed number of cases to the expected number, are identified as potential clusters. The maximum radius of the spatial window was set at 50%, with a Monte Carlo-based p-value <0.05 indicative of statistically significant clusters [60].

#### Cost–distance analysis.

Administrative boundary shapefiles were obtained from the Global Administrative Areas [63]. These shapefiles enabled the extraction of geographic boundaries for Narok County and specifically Narok West Subcounty, which served as the study area. Population distribution data were sourced from WorldPop’s 2020 unconstrained population density estimates for Kenya, which provides gridded population counts at approximately 100-meter resolution [64]. We obtained the walking-only friction surface dataset from the Malaria Atlas Project’s global accessibility mapping initiative, which quantified travel impedance across the study area [65]. This friction surface, generated in January 2020, represents the estimated travel time (in minutes) required to traverse each pixel, accounting for landscape features such as land cover, topography, and water bodies. We cropped both the population and friction surface datasets to retain only the area corresponding to Narok West subcounty. Additionally, we constructed a transition matrix from the friction surface to model movement between adjacent pixels. The transition matrix defined the ease of movement from each pixel to its neighboring pixels and was calculated using the following equation: An eight-directional connectivity pattern was employed [66], allowing movement to all eight surrounding neighbors, which better approximates continuous movement across the landscape, especially in the walking-only scenario. We then calculated the accumulated cost surface using Dijkstra’s algorithm, which determines the minimum least-cost distance from any resistance detection point to all pixels in the landscape, representing the shortest possible travel time from the nearest resistance location to each pixel [67]. The accessibility surface was resampled to match the population's raster resolution of approximately 100 meters at the equator to enable a direct comparison between travel time and population distribution. We categorized the population into four accessibility zones based on walking time to the nearest resistance watering point: ≤ 30 minutes, 30–60 minutes, 60–120 minutes, and >120 minutes. We calculated the proportion of the population within each accessibility category by summing the population values for all pixels within each travel time zone and dividing the total population of the study area was calculated.

## Results

### Prevalence of AMR *E. coli* isolates in surface waters

*E. coli* was isolated in 92.9% (n = 26) of the sampled sites. The two sites where *E. coli* were not detected (sites 12 and 23) were exempted from further analysis. Further seasonal analysis established nuanced *E. coli* encounters (S2 Table), totaling 70 isolates over the study period. Out of the 70 *E. coli* isolates tested, all (100%) were resistant to at least one antibiotic. All isolates were resistant to Penicillin followed by Amoxycillin and Nitrofurantoin at 92.9% and 87.2% respectively (S1 Fig). Gentamicin and Levofloxacin had the highest susceptibility rates at 98.6% followed by Kanamycin (91.4%). Additionally, the study observed a fluctuating resistance pattern of *E. coli* isolates to individual antibiotics over the May 2024 – April 2025 study period (Fig 3). There was statistically significant seasonal variation of AMR *E. coli* (*χ² (6, N = 560) = 13.145, p = 0.04*), with notably higher AMR *E. coli* isolates during the wet seasons (S2 Fig). In relation to AMR variation based on water source types, rivers had higher AMR isolates compared to open water pans (S3 Fig). This variation between water source was statistically significant (*χ²(2, N = 560) = 6.95, p = 0.03*).

**Fig 3:**
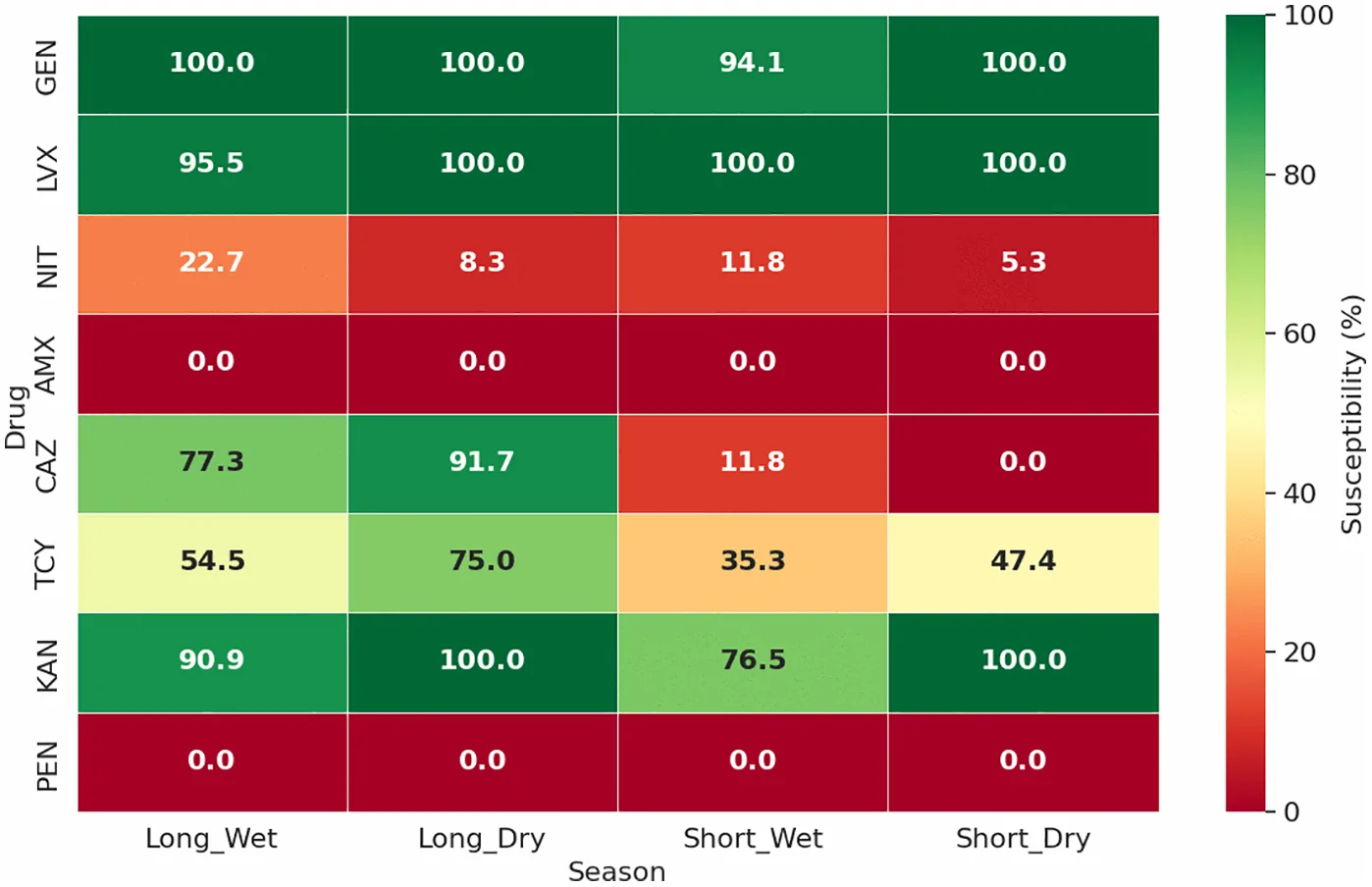
Seasonal antibiotic resistance heatmap in the Narok West surface waters during the study period. GEN = Gentamicin; LVX = Levofloxacin; NIT = Nitrofurantoin; AMX = Amoxycillin; CAZ = Ceftazidime; TCY = Tetracycline; KAN = Kanamycin; and PEN = Penicillin. Caption credit: Waswala-Olewe BM.

### Multi-drug resistance (MDR) scores of surface waters within the Maasai Mara Ecosystem

In reference to the 8 antibiotics tested for multi-drug resistance, five classes of antibiotic categories were tested, i.e., Aminoglycosides, Fluoroquinolones, Nitrofurans, Tetracyclines, and β-Lactams. Majority (80.8%) of the sites had MDR isolates (Table 1), ranging from 3 to 5 antibiotic classes. One site (Site 15) had the highest MDR isolate score of 5 classes, followed by 5 sites (1, 3, 9, 14, 15) at 4 antibiotic classes. 13 sites (2, 4, 5, 7, 11, 13, 16, 17, 18, 21, 26, 27, 28) had an MDR score of three antibiotic classes. The mean MDR score per site sample was 3 antibiotics. Across all the water points, 73.1% of the *E. coli* isolates were MDR. Open water pans had higher MDR *E. coli* isolate rates of 77.8% as opposed to rivers at 62.5%. Furthermore, a chi-square test conducted to determine the MDR relationship between water sources (open water pans and rivers) in antibiotic class 1–5 revealed no significant association (*χ²(4, N = 26) = 4.97, p > 0.05*).

**Table 1:** Predominant antibiotic resistance pattern of *E. coli* isolates from different sampling locations.

Site	GEN	LVX	NIT	AMX	CAZ	TCY	KAN	PEN	Total	MDR
**1**	0	0	3	4	3	3	1	4	18	Yes
**2**	0	0	3	3	2	2	0	3	13	Yes
**3**	0	0	2	2	1	1	1	2	9	Yes
**4**	0	0	2	3	0	1	0	3	9	Yes
**5**	0	0	1	2	0	1	0	2	6	Yes
**6**	0	0	0	2	1	0	0	2	5	No
**7**	0	0	2	3	2	2	0	3	12	Yes
**8**	0	0	3	3	1	0	0	3	10	No
**9**	1	0	2	3	1	1	0	3	11	Yes
**10**	0	0	3	3	2	0	0	3	11	No
**11**	0	0	3	3	2	1	0	3	12	Yes
**13**	0	0	4	4	2	3	0	4	17	Yes
**14**	0	0	3	3	3	3	1	3	16	Yes
**15**	0	1	4	4	3	3	1	4	20	Yes
**16**	0	0	4	4	2	2	0	4	16	Yes
**17**	0	0	2	2	2	2	0	2	10	Yes
**18**	0	0	3	3	3	2	0	3	14	Yes
**19**	0	0	1	1	0	0	0	1	3	No
**20**	0	0	1	2	0	0	0	2	5	No
**21**	0	0	3	3	2	1	1	3	13	Yes
**22**	0	0	3	3	2	0	0	3	11	No
**24**	0	0	1	1	1	0	0	1	4	No
**25**	0	0	2	2	2	2	1	2	11	Yes
**26**	0	0	1	1	0	1	0	1	4	Yes
**27**	0	0	3	3	2	2	0	3	13	Yes
**28**	0	0	2	3	1	1	0	3	10	Yes
**Total**	1	1	61	70	40	34	6	70	283	

Note: Percentages may not sum to 100% due to rounding. GEN = Gentamicin; LVX = Levofloxacin; NIT = Nitrofurantoin; AMX = Amoxycillin; CAZ = Ceftazidime; TCY = Tetracycline; KAN = Kanamycin; and PEN = Penicillin.

In relation to classification by antibiotic classes and the antibiotics we tested, our study revealed that β-Lactams resistance ranked highest (33.8%), followed by Nitrofurans (31.2%), Tetracyclines (24.7%), Aminoglycosides (9.1%) and Fluoroquinolones (1.3%) (Fig 4).

**Fig 4:**
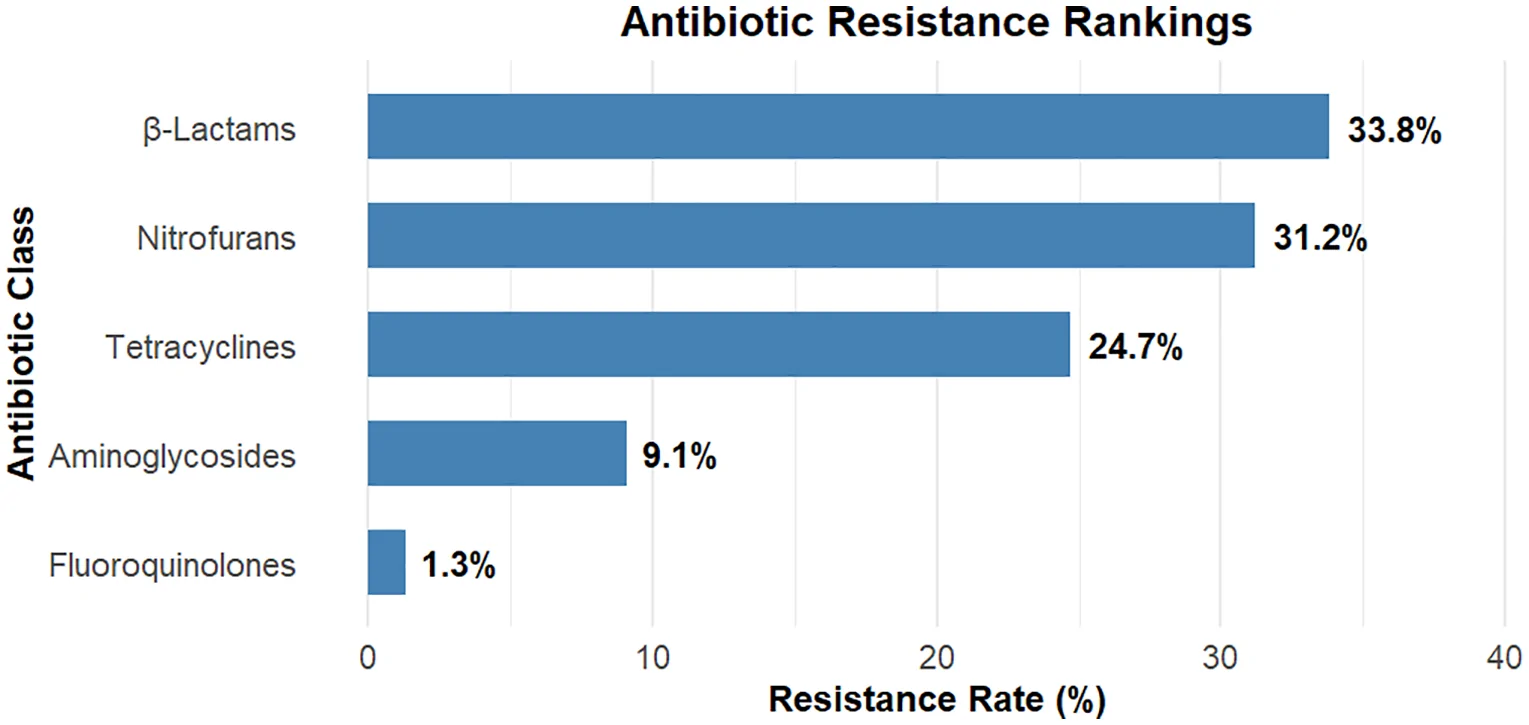
Percentage resistance by antibiotic class in open waters during study period. Caption credit: Waswala-Olewe BM.

The study noted varied levels of *E. coli* isolates phenotypic resistance against 1^st^, 2^nd^ and 3^rd^ generation antibiotics. All isolates were 100% resistant to 1^st^ generation β-Lactams (penicillin) while 1^st^ generation tetracycline class (tetracycline) was 51.5% susceptible. Additionally, the isolates were highly susceptible to second generation aminoglycosides (Gentamicin and Kanamycin at 98.8% and 91.4% respectively) as opposed to second generation aminopenicillin (Amoxicillin) which was 100% resistant. Moreover, 3^rd^ generation antibiotics of classes Fluoroquinolones (Levofloxacin), β-Lactams (Ceftazidime) and Nitrofurans (Nitrofurantoin) had mixed susceptibility rates of *E. coli* from aquatic resources at 98.6%, 42.9% and 12.9%.

### Identification of clustering patterns in AMR profiles

Two exploratory clusters were observed using SaTScan (Fig 5; S3 Table), though these were not statistically significant (p > 0.05). Cluster 1 comprised sampling sites adjacent to high human density areas showed a 0.78 likelihood of relative risk of resistance. Cluster 2 was dominated by sampling sites located along the Maasai Mara National Reserve, exhibiting a 1.26 likelihood relative risk of resistance. This cluster was located near dense human populations sandwiched between protected wildlife conservancies, which are largely considered wildlife dispersal areas.

**Fig 5:**
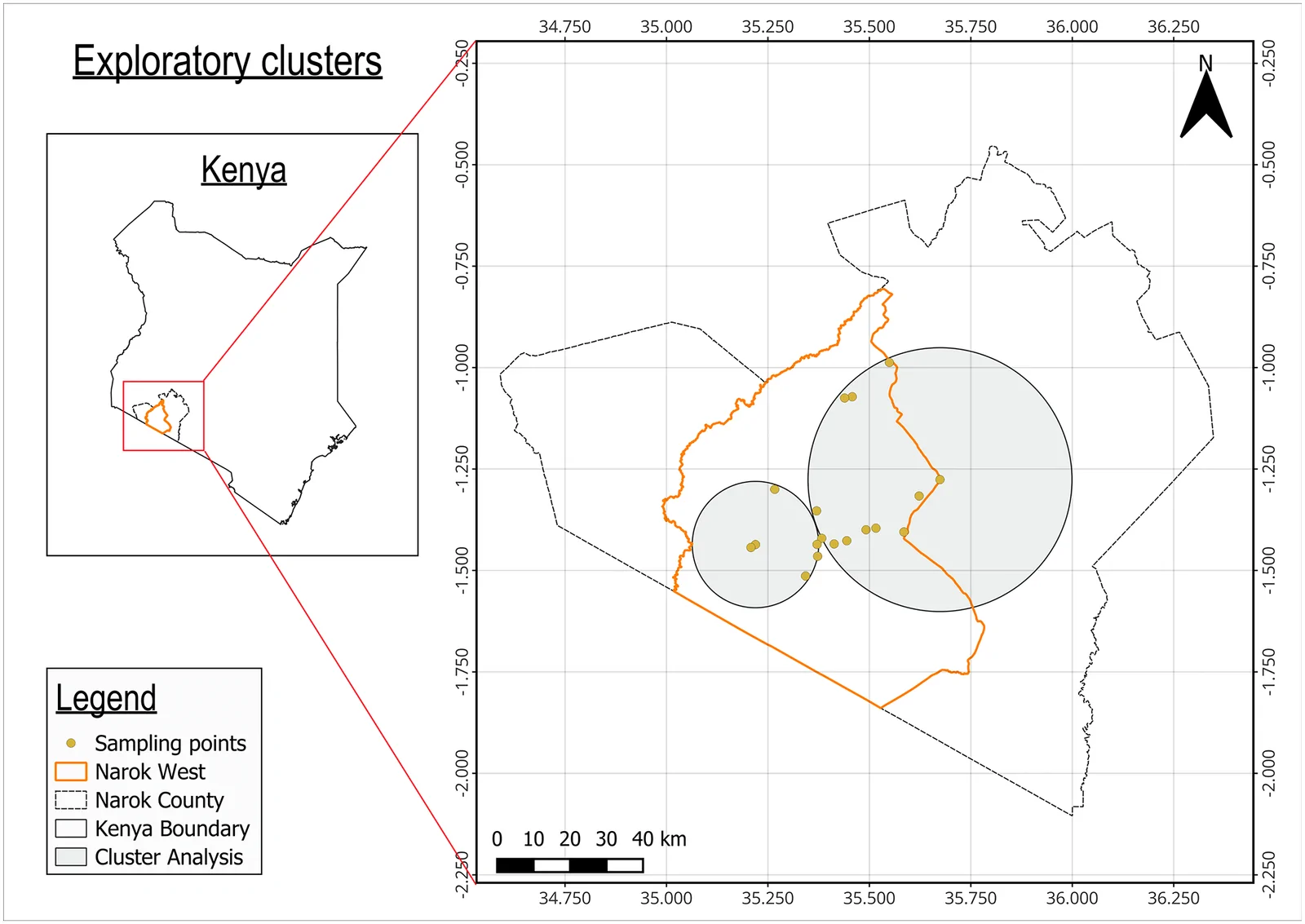
Exploratory clusters derived from the multinomial spatial scan (SaTScan) statistics of *E. coli* AMR profile distribution in the Narok West subcounty during the study period. Cluster 1 included sampling sites adjacent to areas of high human density, while Cluster 2 was dominated by sampling sites located along the Maasai Mara National Reserve, where dense human populations are sandwiched between wildlife conservancies. None of the clusters were statistically significant (p > 0.05). Caption credit: Waswala-Olewe BM.

### Cost-distance analysis

Using cost-distance analysis, we established that at least a third of the MME population is at risk of encountering AMR *E. coli* isolates from surface water sources (Figs 6; S4 Table).

**Fig 6:**
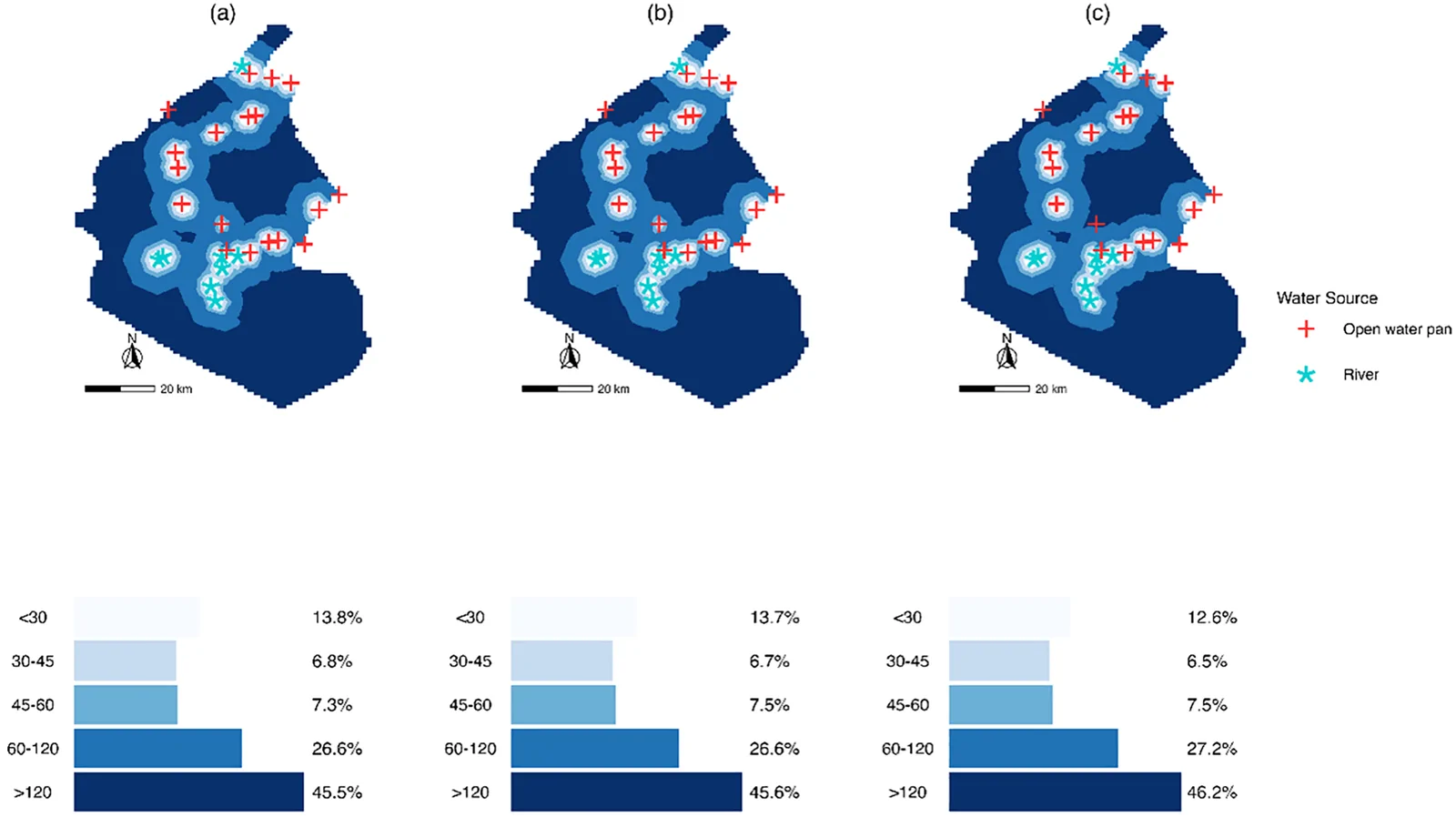
Cost-Distance analysis during the study period. 6A All seasons. 6B Wet seasons analysis. 6C Dry season analysis. Caption credit: Waswala-Olewe BM.

## Discussion

Our study aimed to characterize the prevalence of antimicrobial resistance, resistance patterns, and seasonal variations of *E. coli* isolates from surface water sources, as well as to assess the spatial distribution of resistance profiles in the Maasai Mara Ecosystem. The study is the first to confirm the presence of AMR and MDR isolates in the multi-use surface waters of Kenya’s ASALs. These findings align with previous studies that identified AMR *E. coli* isolates exist in water sources across various landscapes [68,69]. The study also established above-average MDR scores against conventional antibiotics, i.e., over 70% of the isolates encountered were MDR. The high AMR and MDR *E. coli* isolates in multi-use freshwater sources within the landscape are potentially emerging public and One Health Quadripartite concern [70,71], especially with weather variation and climate change [37–39,72].

SaTScan revealed two exploratory clusters. Cluster 1 comprised sampling sites adjacent to high human density areas, and an *E. coli* isolate from any of these sites was less likely to be resistant (relative risk = 0.78), compared to an isolate from any other sampling site in the ecosystem. In comparison, Cluster 2 was dominated by sampling sites located along the Maasai Mara National Reserve, with dense human populations sandwiched between protected wildlife conservancies, which are largely considered wildlife dispersal areas. An *E. coli* isolate from any of these sampling sites showed a higher relative risk of resistance (RR = 1.26), although this cluster was not statistically significant, the finding may reflect chance variation given the limited number of isolates within clusters compared to those from other sampling sites. As neither cluster reached statistical significance, these findings are exploratory and hypothesis-generating rather than confirmatory, and are best interpreted as suggestive of a possible higher rate of AMR *E. coli* in water sources in proximity to human settlements and are influenced by anthropogenic activities [73–75]. These findings of elevated prevalence of antimicrobial-resistant *E. coli* isolates in water sources near human settlements probably confirm that human activities influence distribution and resistance of *E. coli* isolates based on poor/limited use and access to water, sanitation, and hygiene (WASH) facilities [26]; livestock movement, transhumance and corralling of people as a result of wildlife conservancy establishment [43,76,77]; poor use of antimicrobials and stewardship [16,20,78] in hope of managing / treating endemic livestock diseases [19], poor antimicrobial use / stewardship within the shared human-livestock-wildlife landscape [52,77], and discharge from wastewater treatment plants located upstream. This is the first study to our knowledge that characterized AMR *E. coli* prevalence and temporal distribution in a shared multi-use open water pans dominated landscape in East Africa.

Moreover, with Narok West subcounty being located within the Mara–Serengeti landscape, a prominent tourist destination [40], there exists a significant risk of travel-related *E. coli* transmission [41,42]. We, however, note that since there are SaTScan AMR clusters, the results necessitate further assessments on the prevalence of true clustering phenomena of AMR *E. coli* isolates in the study area, especially since this study cluster findings are baseline. Our study could have plausibly identified an emerging concern regarding the use of river and open water pan waters for horticultural irrigation. This practice may not only increase the risk of fecal-oral transmission of waterborne pathogens, particularly via the ingestion of raw, inadequately washed, or undercooked fruits and vegetables [79–81] but also ingestion of AMR isolates.

Cost-distance analysis revealed that at least a third of the MME population is at risk of encountering AMR *E. coli* isolates from open-access and shared multi-use surface water sources within the water-scarce landscape. This is attributed to limited access to potable water, as surface water points are the primary water sources. These findings are of concern since surface waters are the primary water sources, with most humans and animals converging to drink and drawing domestic water from them [25,26]. Poor/limited access to potable water can result in direct consumption of this water with a high presence of AMR and MDR *E. coli* isolates [3,26], resulting in increased severe gastrointestinal infections and potential fatalities [4,82,83]. Additionally, due to weather and seasonal variations [72], the available water bodies can become conduits of AMR gene transfer [84,85], particularly during seasonal flooding [86,87]. These floods can potentially increase deleterious AMR and MDR *E. coli* isolates in rivers and open water pans, especially within downstream landscapes, thus undermining public and ecosystem health.

The rising resistance to essential antibiotics, as identified by WHO and WOAH, constitutes a significant public health issue and a One Health challenge. Human antibiotics susceptibility scores based on WHO ranking and dosages were as follows: highest-priority critically important antibiotics (LVX_5, 1.4%; CAZ_30, 57.2%); critically important antimicrobics (GEN_10, 1.4%; KAN_30, 8.6%; AMX_30, 100%); highly important antimicrobics (TCY_30, 48.6%); and important antimicrobics (NIT_300, 87.1%). The high resistance to Ceftazidime at 57.2%, third-generation broad-spectrum cephalosporin antibiotic classified as highest-priority critically important antibiotic is of public health concern. In addition to Ceftazidime resistance, Levofloxacin and Gentamycin resistance warrant concern especially due to transmission of antibiotic resistance genes by water bodies [87,88]. Further analysis revealed that all 42 out of 70 (60%) *E. coli* isolates showed a resistance pattern suggestive of Extended Spectrum β-lactamase (ESBL) production [89]. ESBLs are enzymes that hydrolyze third generation cephalosporins and are usually inhabited by clavulanic acid. Ceftazidime exhibited high resistance across all seasons as opposed to Gentamicin. The high resistance to Ceftazidime in comparison to Gentamicin plausibly suggests stable ESBL type resistance in surface water bodies throughout the study period. This pattern is consistent with phenotypic ESBL [90] indicating likely hydrolysis of third-generation cephalosporins and reduced effectiveness of β-lactam antibiotics. The increased resistance may lead to higher morbidity and mortality from waterborne diseases, as the effectiveness of the prescribed medication could be diminished or more costly.

From an ecological perspective, we established high AMR and MDR *E. coli* isolates in the open surface water sources of Narok West subcounty. These levels can potentially create selective pressures on natural bacterial strains, which includes killing susceptible isolates while increasing resistant isolates and their antimicrobial resistance genes [91–94]. Additionally, antibiotic metabolites in water cause genotoxicity, histopathological changes, and alterations in fish development, metabolic and cardiovascular systems [95,96]; disrupt and alter intra- and interspecific plankton associations [97]; and impair growth and reproduction of aquatic invertebrates and fish while disrupting primary production in the form of photosynthesis [98], thus altering ecological resilience and fishing livelihoods.

### Evidence gaps/study limitations

Sources of microbial pollution, colony counts and physicochemical parameters of sampled water bodies were beyond the scope of this study. Since the ecosystem is quite extensive coupled by paucity of data of number of OWPs and limited resources (finances, time, personnel and equipment), this study sought to establish baseline data on AMR *E. coli* isolates in surface water bodies and recommends further studies. Additionally, we were unable to establish pathogen contamination loads based on water dilution and locations within waterpoints (shoreline vs center of river / OWP). Finally, we could not establish which antibiotics were most accessible in the region, nor if human and livestock antibiotic usage patterns contributed to the AMR and MDR findings.

## Conclusions

Our study establishes and confirms the presence of AMR and MDR *E. coli* colonies in a water-scarce landscape in Kenya, where shared multi-use surface waters are the primary sources of water. The presence of the AMR and MDR isolates can serve as facilitating zones for the interaction and spread of AMR colonies, which may increase the risk of contamination exposure for local human and animal populations and the environment at large. The situation is aggravated by inadequate access to WASH infrastructure [26], seasonal environmental changes, and weather patterns that result in flash floods, which bring contaminated water from wastewater treatment facilities. Additionally, shifting weather and climate change directly lead to the drying of temporary open water pans [72]. The seasonal fluctuations of surface waters can result in the transport of AMR strains to downstream communities and landscapes, prompting transhumance and wildlife migration. This process may facilitate the introduction and colonization of these AMR colonies in pristine areas via surface run-offs, floods, and population movement. These findings highlight the urgent need to improve access to WASH infrastructure in Narok West subcounty, enhance public health through certified and accredited human and veterinary health practitioners, raise community awareness about AMR via information, education, and communication, conduct seasonal surveys on AMR contamination levels in surface waters along with microbial source tracking, and perform genotypic identification of AMR strains and genes using genome sequencing and bioinformatic analysis. Such an effort is critical, as presently there are limited testing programmes of AMR levels in ASAL and shared landscapes. Our study not only demonstrated the benefit of spatial and temporal clustering analysis using SaTScan to identify and map exploratory clusters of AMR isolates in the fragile Maasai Mara Ecosystem but also champion the use of WHONET in environmental AMR surveillance, in conformity to the “Quadripartite” alliance. The exploratory, non-significant spatial clustering of AMR *E. coli* isolates in water sources in proximity to human settlements nonetheless underscores the importance of further investigating anthropogenic drivers of AMR through a One Health lens.

### Recommendation

Using a blend of system thinking and the One Health Quadripartite Approach, there is an urgent need to raise awareness of AMR and its impacts while advocating for stringent antibiotic stewardship and evidence-based guidelines. Based on the AMR and MDR isolates present in primary water sources, relevant water provision and safety institutions should double their efforts in the provision of potable water. Local communities should be encouraged to fetch water from dedicated areas, such as constructed wells, in situations where access to and provision of potable water is dire. These should be complemented by potable water treatment. Public health official should encourage adoption of water, sanitation and hygiene practices (avoid open defecation) while also reducing animal related point and non-point pollution. In relation to ecosystem health, we propose increased periodic surveillance and early warning of AMR and MDR contamination in surface waters; phylogeographic and metagenomic analysis of AMR isolates among other ubiquitous waterborne pathogens; microbial and AMR *E. coli* pollution source tracking; and reporting in WHO’s Global Antimicrobial Resistance Surveillance System (GLASS) and WOAH’s Animal Antimicrobial Use (ANIMUSE). Additionally, our study proposes further research on the spread of AMR networks, considering the seasonal transhumance of livestock and wildlife migration. Finally, we advocate for studies on socio-cultural anthropology including knowledge, attitudes, and perceptions regarding antibiotic use, as well as the efficacy of antibiotics and AMR on community health and livelihoods.

### Importance of study

This study results revealed prevalence and seasonal variation of AMR *E. coli* isolates within shared multi-use water sources in ASAL Kenya and SSA, their antibiotic resistance and multiple antibiotic resistance indices, and cluster patterns of the antimicrobial resistance profiles. This study helps fill the data dearth on seasonal environmental AMR in Kenya and spatial clusters. The sampling locations, sources of water samples and seasonality of sampling contribute to informing how AMR in the environment might be influenced in shared human-livestock-wildlife ecosystems, especially ASALs.

## Supporting information

S1 Table Sampling sites metadata. (DOCX)

S2 Table Seasonal *E. coli* encounter rates in multi-use surface waterpoints over the study period. (DOCX)

S3 Table SaTScan clustering of antimicrobial resistance profiles distribution in Narok West subcounty over the study period. (DOCX)

S4 Table Cost-distance analysis. (DOCX)

S1 Fig Overall phenotypic antibiotic resistance summary in Narok West surface waters over the study period. (TIFF)

S2 Fig Seasonal phenotypic resistance pattern of *E. coli* isolates within open water pans and rivers during the study period. (TIFF)

S3 Fig Resistance profiles based on water sources during the study period. (TIFF)
