# Supplementary material for: Spatiotemporal patterns of antimicrobial resistance in shared human-animal waterpoints in the Maasai Mara Ecosystem, Kenya
Source: PLOS Glob Public Health. 2026 Oct 7;6(10):e0007393. doi: 10.1371/journal.pgph.0007393 (PMC13645049; doi:10.1371/journal.pgph.0007393)
Supplement: Supplementary file 1 [file pgph.0007393.s001.docx]

Supplementary Table 1. Sampling sites metadata during study period

| **Sampling site** | **Latitude** | **Longitude** | **Water body** | **Water use and anthropogenic activity around** | **Presence of faecal matter** |
| --- | --- | --- | --- | --- | --- |
| 001 | - 0.9427 | 35.4238 | River | Domestic water collection, car washing, irrigation and livestock watering | Yes (livestock) |
| 002 | - 0.9636 | 35.4416 | Open water pan | Domestic water collection, irrigation, and livestock watering | Yes (wild birds and livestock) |
| 003 | - 0.9748 | 35.5002 | Open water pan | Crops farming, irrigation and livestock watering | Yes (livestock) |
| 004 | - 0.9873 | 35.5493 | Open water pan | Irrigation and livestock watering | Yes (wild birds and livestock) |
| 005 | - 1.0718 | 35.4578 | Open water pan | Domestic water collection and livestock watering | Yes (wild birds and livestock) |
| 006 | - 1.0749 | 35.4390 | Open water pan | Livestock watering | Yes (livestock) |
| 007 | - 1.1156 | 35.3566 | Open water pan | Domestic water collection and livestock use | Yes (livestock) |
| 008 | - 1.0563 | 35.2313 | River | Livestock watering | Yes |
| 009 | - 1.1673 | 35.2499 | Open water pan | Livestock watering | Yes (livestock) |
| 010 | - 1.2072 | 35.2568 | Open water pan | Livestock watering and wildlife use (watering and conservation) | Yes (wild birds and wildlife) |
| 011 | - 1.3001 | 35.2667 | Open water pan | Livestock watering and wildlife use (watering and conservation) | Yes (wild birds and wildlife) |
| 012 | - 1.2985 | 35.6465 | Open water pan | Livestock watering and wildlife use (watering and conservation) | Yes (wild birds and wildlife) |
| 013 | - 1.4361 | 35.2193 | River | Laundry, livestock watering and wildlife conservation | Yes (wild birds and wildlife) |
| 014 | - 1.4433 | 35.2082 | River | Laundry, water collection, bathing and livestock watering | Yes (livestock) |
| 015 | - 1.5136 | 35.3427 | River | Laundry, water collection, swimming, bathing and livestock watering | Yes (livestock) |
| 016 | - 1.5032 | 35.3548 | Open water pan | Livestock watering | Yes (livestock) |
| 017 | - 1.4650 | 35.3723 | River | Sand harvesting, laundry, water collection, bathing and livestock watering | Yes (livestock) |
| 018 | - 1.4358 | 35.3702 | River | Livestock watering and wildlife conservation | Yes (elephants and livestock) |
| 019 | - 1.3531 | 35.3699 | Open water pan | Wildlife use (watering and conservation) | Yes (wild birds and wildlife) |
| 020 | - 1.4208 | 35.3827 | Open water pan | Wildlife use (watering and conservation) | Yes (human, wild birds and wildlife) |
| 021 | - 1.4346 | 35.4131 | River | Wildlife use (watering and conservation) | Yes (wild birds and wildlife) |
| 022 | - 1.4267 | 35.4442 | Open water pan | Wildlife use (watering and conservation) | Yes (wild birds and wildlife) |
| 023 | - 1.4162 | 35.4548 | Open water pan | Irrigation, livestock watering and wildlife use (watering and conservation) conservation | Yes (wild birds and wildlife) |
| 024 | - 1.3997 | 35.4917 | Open water pan | Irrigation, livestock watering and wildlife use (watering and conservation) conservation | Yes (wild birds and wildlife) |
| 025 | - 1.3957 | 35.5161 | Open water pan | Livestock watering and wildlife use (watering and conservation) conservation | Yes (wild birds and wildlife) |
| 026 | - 1.4050 | 35.5849 | Open water pan | Irrigation, livestock watering and wildlife use (watering and conservation) conservation | Yes (wild birds and wildlife) |
| 027 | - 1.3164 | 35.6225 | Open water pan | Livestock watering and wildlife use (watering and conservation) conservation | Yes (wild birds and wildlife) |
| 028 | - 1.2817 | 35.6729 | Open water pan | Livestock watering and wildlife use (watering and conservation) conservation | Yes (wild birds and wildlife) |
