# Supplementary material for: Spatiotemporal patterns of antimicrobial resistance in shared human-animal waterpoints in the Maasai Mara Ecosystem, Kenya
Source: PLOS Glob Public Health. 2026 Oct 7;6(10):e0007393. doi: 10.1371/journal.pgph.0007393 (PMC13645049; doi:10.1371/journal.pgph.0007393)
Supplement: Supplementary file 2 [file pgph.0007393.s002.docx]

Supplementary Table 2. Seasonal E. coli encounter rates in multi-use surface waterpoints during study period

| **Sampling site** | **Season 1** | **Season 2** | **Season 3** | **Season 4** |
| --- | --- | --- | --- | --- |
| 001 | + | + | + | + |
| 002 | + | ND | + | + |
| 003 | + | ND | + | ND |
| 004 | + | + | + | ND |
| 005 | + | + | ND | ND |
| 006 | + | ND | ND | + |
| 007 | + | ND | + | + |
| 008 | + | + | ND | + |
| 009 | + | + | + | ND |
| 010 | + | + | ND | + |
| 011 | + | ND | + | + |
| 012 | ND | ND | ND | ND |
| 013 | + | + | + | + |
| 014 | + | ND | + | + |
| 015 | + | + | + | + |
| 016 | + | + | + | + |
| 017 | ND | ND | + | + |
| 018 | + | ND | + | + |
| 019 | + | ND | ND | ND |
| 020 | + | + | ND | ND |
| 021 | + | ND | + | + |
| 022 | ND | + | + | + |
| 023 | ND | ND | ND | ND |
| 024 | ND | ND | ND | + |
| 025 | + | ND | ND | + |
| 026 | ND | + | ND | ND |
| 027 | + | ND | + | + |
| 028 | + | ND | + | + |
| **Total encounters** | **22** | **12** | **17** | **19** |

+ = encountered

ND = not detected
