# Supplementary material for: Spatiotemporal patterns of antimicrobial resistance in shared human-animal waterpoints in the Maasai Mara Ecosystem, Kenya
Source: PLOS Glob Public Health. 2026 Oct 7;6(10):e0007393. doi: 10.1371/journal.pgph.0007393 (PMC13645049; doi:10.1371/journal.pgph.0007393)
Supplement: Supplementary file 3 [file pgph.0007393.s003.docx]

Supplementary Table 3. SaTScan clustering of AMR profiles distribution in Narok West subcounty during study period.

| Cluster | Radius | Sampling Site | Category | Number in Cluster (%) | Relative Risk |
| --- | --- | --- | --- | --- | --- |
| 1 | 36.13km | 4, 5, 6, 19, 20, 21, 22, 24, 25, 26, 27, 28 | Susceptible | 54.8 | 1.18 |
|  |  |  | Intermediate | 7.2 | 1.41 |
|  |  |  | Resistant | 38.0 | 0.78 |
| 2 | 17.30km | 11, 13, 14, 15, 17, 18 | Susceptible | 41.4 | 0.79 |
|  |  |  | Intermediate | 5.9 | 1.01 |
|  |  |  | Resistant | 52.6 | 1.26 |

*All clusters were not statistically significant (p>0.05).*
