# Supplementary material for: Spatiotemporal patterns of antimicrobial resistance in shared human-animal waterpoints in the Maasai Mara Ecosystem, Kenya
Source: PLOS Glob Public Health. 2026 Oct 7;6(10):e0007393. doi: 10.1371/journal.pgph.0007393 (PMC13645049; doi:10.1371/journal.pgph.0007393)
Supplement: Supplementary file 4 [file pgph.0007393.s004.docx]

Supplementary Table 4. Cost-distance analysis during study period

| Season | Travel time (minutes) | Population | % of the population |
| --- | --- | --- | --- |
| All seasons | >120 | 82,200 | 45.53 |
|  | 60-120 | 47,977 | 26.57 |
|  | 45-60 | 13,146 | 7.28 |
|  | 30-45 | 12,252 | 6.79 |
|  | <30 | 24,981 | 13.84 |
| Wet season | >120 | 82,285 | 45.58 |
|  | 60-120 | 48,040 | 26.61 |
|  | 45-60 | 13,539 | 7.50 |
|  | 30-45 | 12,037 | 6.67 |
|  | <30 | 24,645 | 13.65 |
| Dry season | >120 | 83,449 | 46.22 |
|  | 60-120 | 49,107 | 27.20 |
|  | 45-60 | 13,458 | 7.45 |
|  | 30-45 | 11,711 | 6.49 |
|  | <30 | 22,830 | 12.64 |
